# Memoirs of the Royal Academy of Sciences at Paris

**Published:** 1781-09

**Authors:** 


III. *Memoirs of the Royal Academy of Sciences at Paris.* *(Continued from page* 110.*)*

VI. *THIRD memoir on the gritt ſtones of Fontainebleau, or an analyſis of thoſe ſtones, and particularly of chryſtallized gritts, by* M. de Laſſone.

VII. *Experiments on the aſhes employed by the manufacturers of ſalt petre at Paris; with remarks on their uſe, by* M. Lavoiſier.—Theſe people it ſeems make uſe of aſhes deprived of their vegetable alkali by repeated waſhing. M. Lavoiſier examines the reaſon why theſe aſhes are preferred to potaſh, and he finds it is becauſe they contain a ſmall portion of muriatic ſalt.

VIII. *Expe-*

VIII. *Experiments on animal respiration, and on the changes produced in the air by its passage through the lungs, by* the same.——Of all the phenomena of the animal œconomy, there are none more deserving the attention of physiologists, than those which accompany respiration. Dr. Priestley has observed, that this process has the property of phlogisticating air in the same manner as the calcination of metals and other chemical processes do; and that it ceases to be fit for respiration when surcharged or saturated with phlogiston. The author of the paper before us is of opinion, that in respiration while one portion of the vital air, as he terms it, is absorbed by the lungs, another portion is converted into fixed air, or what, in imitation of the late M. Bucquet, he calls chalky aeriform acid (*acide crayeux aeriforme*). He has been led to this opinion by the following observations:—1. In calcining mercury in a given portion of atmospheric air fit for respiration, part of this air unites with the mercury, and the remainder ceases to be fit either for respiration or combustion. It no longer diminishes with nitrous acid, but it does not render lime water turbid. If by a violent heat the air is again separated from the calcined mercury, it restores to the other air all its pro-

 perties.

perties. —2. If an animal dies under the receiver of an air pump, the air contained in the veſſel is rendered unfit for combuſtion. It does not diminiſh with nitrous acid, but it decompoſes lime water; an eſſential difference this between this reſiduum and that produced by the calcination of mercury.—3. If cauſtic alkali is placed in this air, part of the alkali loſes its cauſticity, and abſorbs a portion of the air. This new reſiduum of air deprived by animal reſpiration of the vital air it contained, and by cauſtic alkali of its fixed air, is in no reſpect different from the air that remains after the calcination of mercury, and the addition of air diſengaged from the mercury is ſufficient to render it fit for animal life. Hence our author concludes, that while the calcination of mercury ſeems to affect atmoſpherical air merely by ſeparating from it its vital air, or in other words, that portion of it which is eſſential to life, reſpiration ſeems, beſides this ſame effect, to generate in the atmoſpherical air a portion of fixed air.

IX. *Obſervations on the combuſtion of candles in atmoſpherical air, and in air eminently reſpirable, by* the ſame.—By air eminently reſpirable, our author means that which Dr. Prieſtley (in his opinion) improperly terms dephlogiſticated air. He

He ſuppoſes the air we breathe to be compoſed of about one fourth of this pure air, and that the remaining three fourths are a mephitic air, the nature of which is as yet wholly unknown, but which is unequal to the ſupport of animal life, or of combuſtion.

He concludes from his experiments, 1ſt, That this mephitic portion of atmoſpherical air does not in any degree contribute to the phenomena of combuſtion. 2dly, That combuſtion acts only on the portion of air eminently reſpirable. 3dly, That two-fifths of this pure air are converted into fixed air by the burning of candles, and that the remaining three-fifths unite with the mephitic portion in ſuch a manner as to be inſeparable by combuſtion. 4thly, That phoſphorus has much greater combuſtible powers than candles, ſince it is able to exhauſt four-fifths of the pure air contained in atmoſpherical air. 5thly, That pyrophorus is ſtill more powerful in this reſpect, being able to exhauſt almoſt the whole of the pure part.

In a future paper the author engages to prove the exiſtence of the matter of fire in all aeriform fluids, and to ſhew how fixed air may be formed by combining inflammable air with the baſis of air eminently reſpirable.

X. *Re-*

X. *Remarks on the neceſſity of performing the Cæſarean operation on women who die in advanced pregnancy, and on the means of reſtoring their children to life when apparently dead, by* M. Bordenave.— There are not wanting inſtances to prove, that the *fœtus in utero* oftentimes ſurvives its mother when ſhe dies during pregnancy, and of courſe may be ſometimes ſaved by the Cæſarean ſection. Cangiamila, in his *Embryologia Sacra*, has collected a great number of inſtances in which it was performed with ſucceſs. He obſerves, that in Montreal and its neighbourhood, in the ſpace of about 24 years, 21 children had been preſerved in this manner; that from 1704 to 1748, of 60 children taken out in this way at Caltaniſſecta only five died, and at Victoria in the dioceſe of Syracuſe twenty, all of whom lived. To theſe facts from Cangiamila our author adds one of the ſame kind, communicated to him from the neighbourhood of Mont de Marſan. A woman in her ſixth month died at one o'clock in the afternoon, and was opened at ſeven in the evening. The child was reſtored and lived upwards of two hours. Cangiamila ſpeaks of five inſtances in which the lives of the children were preſerved though the operation was performed 15, 23, 24, and even more hours after the death of the mother.

ther. In these cases M. Bordenave recommends the usual methods of recovery, and amongst others the bathing the child in an aromatic decoction and the sprinkling it with cold water.

XI. *An account of an aeriform substance that proceeds from the human body, and on the means of collecting it, by* Count de Milly.

XII. *Second Memoir on the same subject, by* the same.

Our author, while in the warm bath, perceived small bubbles of air forming on different parts of his body and rising to the surface of the water. He collected a small quantity of this air, which he examined with M. Lavoisier, and they found that it extinguished flame and rendered lime water turbid.

XIII. *Experiments to prove that the acid of phosphorus procured, according to Mr. Scheele's method, from calcined bones, is not a pure acid, by* M. Sage.—By employing Mr. Scheele's process for procuring the phosphoric acid, that acid is not obtained pure, but in the form of a vitreous mass which is insoluble in water. This substance when distilled with charcoal yields phosphorus, and the proportion of phosphoric acid formed, is found to be about a fourth part of the vitreous mass. M. Sage has not yet been able to determine mine precisely what the substance is, which by uniting with the acid gives it the vitreous appearance, or to ascertain whether this matter is a real glass or a saline substance.

XIV. *Observations on the solution of mercury in vitriolic acid, and on the resolution of this acid into aeriform sulphureous acid and air eminently respirable, by* Mr. Lavoisier.—According to this writer sulphur is vitriolic acid deprived of vital air, which is one of its principles. When a solution of mercury in vitriolic acid is distilled, it yields an acid resembling fixed air, after this, air eminently respirable rises, and then the mercury resumes its original form.

XV. *Experiments on the combination of alum with combustible substances, and on the changes produced in the air by the burning of pyrophorus, by* the same.—We know that pyrophorus is a kind of liver of sulphur, which has the property of inflaming spontaneously in the open air. By burning it in close vessels filled with common air M. Lavoisier found that it burned till it had absorbed somewhat more than a fourth part of the common air. The remainder of the air evidently contained fixed air. By burning pyrophorus in air eminently respirable it destroyed about six-sevenths. He observes, that if the fixed air which which this residuum contains is separated, by passing it through water, we procure a new and very pure air in which pyrophorus will burn, and that by repeating this operation he has been able to combine with the pyrophorus the whole of the vital air, to within about 144th part. The pyrophorus had increased in weight by burning, and was no longer a hepar sulphuris; but was of a white colour, and had the astringency and all the other properties of alum.

XVI. *Remarks on the vitriolization of martial pyrites*, *by* the same. Martial pyrites are a combination of iron and sulphur. Distilled in their natural state they yield sulphur; but they undergo a change when exposed to the air, and if washed after this yield vitriolum martis. M. Lavoisier has endeavoured to ascertain how the air acts in their decomposition, and finds that it is by the pyrites absorbing that part of the vital air contained in the atmospherical air employed in the operation.

XVII. *Of the combination of the matter of fire with evaporable fluids, and of the formation of elastic aeriform fluids*, *by* the same. It has long been known that evaporation produces cold. M. Lavoisier finds by experiments, that this effect takes place when the fluid is evaporated *in*

 *vacuo*.

*vacuo*. He ſuppoſes, that the matter of fire exiſts in bodies in two different ſtates, being either free, and ſerving to diſſolve bodies, or at leaſt to dilate them, or combined with them and forming one of their principles. In all the operations that abſorb a part of this free fire *(feu libre)* as our author calls it, cold is produced; in all thoſe in which it is diſengaged, heat. He concludes, that every elaſtic aeriform fluid is a combination of the matter of fire with any fluid or even ſolid volatile body; and that volatility is no other than the property bodies have of diſſolving in a certain manner, and of uniting with the matter of fire and forming with it aeriform fluids.

XVIII. *Obſervations on the phoſphoric acid obtained by the deliquium of phoſphorus, and on the neutral ſalts which reſult from a combination of this acid with alkalis, by* M. Cadet.—An ounce of phoſphorus furniſhes, by *deliquium*, three ounces of an acid that has neither colour nor ſmell. Combined with fixed alkalis or abſorbent earths it forms a neutral ſalt which is not deliqueſcent. Theſe experiments prove, that the acid obtained in this manner is different from that which is procured by deflagration.

XIX. *Obſervations on the acid of ſugar, by the* ſame—This is an account of Profeſſor Bergman's man's proceſs, which our readers will find deſcribed in the Firſt Volume of our Journal, page 81.

[ *To be concluded in our next.* ]

