# Errata

**Published:** 1781-12

**Authors:** 


					?p. 179, 1. 44, ar.d
p. 180, 1. 4, for ' nitrous aid' read ' nitrous air.'?

				

